# Clinical outcomes of de novo metastatic HER2-low breast cancer: a National Cancer Database Analysis

**DOI:** 10.1038/s41523-022-00498-8

**Published:** 2022-12-30

**Authors:** Changchuan Jiang, Stuthi Perimbeti, Lei Deng, Charles L. Shapiro, Shipra Gandhi

**Affiliations:** 1 Department of Medicine, Roswell Park Comprehensive Cancer Center, Buffalo, NY USA; 2 Division of Hematology and Medical Oncology, Department of Medicine, Icahn School of Medicine at Mount Sinai, New York, NY USA

**Keywords:** Breast cancer, Prognostic markers, Cancer epidemiology, Breast cancer

## Abstract

The development of novel anti-HER2 drugs opens new treatment options for women with breast cancers, including lower expression of HER2. The epidemiology and clinical outcome of metastatic HER2-low breast cancer remain not well described. We designed a retrospective cohort study of the 2010–2017 National Cancer Database (NCDB) was designed to compare the overall survival of HER2-low and HER2-zero de novo metastatic breast cancer with systemic therapy. Multivariable Cox regression models were performed to estimate hazard ratios (HR), adjusting for sociodemographic and clinical factors. A total of 20,636 of 30,929 (66.7%) patients were HER2-low and 10,293 (33.3%) were HER2-zero. When stratified by hormonal receptor status, HER2-low tumors account for 18,066 (69.7%) cases in HR+/HER2− population and 2570 (51.4%) cases in HR−/HER2− population. The prevalence of HER2-low tumors was similar across racial groups with a slightly lower prevalence among the Hispanic population. Women with HER2-low tumors had longer overall survival (OS) than women with Her2-zero tumors in both HR-positive (median OS 39.0 months vs. 37.1 months; adjusted HR: 0.95, 95%CI (0.91–0.98)) and HR-negative groups (median OS 15.8 months vs. 14.1 months; adjusted HR: 0.92 95%CI (0.86–0.98)). The survival advantage was primarily observed in patients who received chemotherapy as their first line of treatment (HR 0.92 95%CI (0.88–0.96) vs. 0.99 95%CI (0.94–1.04), *p*-interaction = 0.04). In summary, HER2-low tumors, irrespective of hormone receptor status, have better survival than HER2-zero tumors in the de-novo metastatic setting. The survival advantage was primarily observed in patients who received chemotherapy in the first line.

## Introduction

HER2 is a critical oncogene and well-established therapeutic target in breast cancer and other cancers^1^. Over the past 20 years, anti-HER2 therapies such as trastuzumab, pertuzumab, lapatinib, trastuzumab-emtansine (T-DM1), trastuzumab deruxtecan (T-DXd), margetuximab, neratinib, and tucatinib revolutionized the treatment of metastatic HER2-overexpressing breast cancers. Currently, the median overall survival in HER2-overexpressing breast cancer has been improved to approximately 5 years^2,3^

Despite the significant improvements in treatments, the benefit of anti-HER2 therapies is confined to a particular group of tumors with HER2 overexpression (IHC3+) or in situ hybridization [ISH]-positive)^4^. Conventional anti-HER2 targeted treatment did not achieve similar success in HER2-negative diseases, including tumors with a low HER2 expression (IHC1+ or IHC2+/ISH-negative)^5^.

Recently, a few new HER2-directed antibody conjugates (ADCs), namely trastuzumab deruxtecan (T-DXd), trastuzumab duocarmazine (SYD985), disitamab vedotin (RC48-ADC), and MRG002 have promising activity in breast cancer with low HER2 expression in early phase clinical trials^6–11^. DESTINY-Breast04 later confirmed T-DXd to be the new standard of care in pretreated breast cancer with low HER2 expression with remarkable improvement in both progression-free survival and overall survival^12^.

While HER2-low disease is gaining increasing attention, high-quality data are lacking regarding the clinical outcomes of metastatic HER2-low expression breast cancers^7^. A few single-center or regional observational studies yielded conflicting results. These studies only included a limited population with metastatic breast cancer^13–18^. In addition, these data may not represent the US patient population^14–16,18^. Therefore, this study examines and compares the clinical outcome of de novo metastatic HER2-low (IHC1+ or IHC2+/ISH-Negative) and HER2-zero (IHC 0) breast cancer using the NCDB, a national cancer outcome dataset in the United States.

## Results

### Patients characteristics

**Fig. 1:**
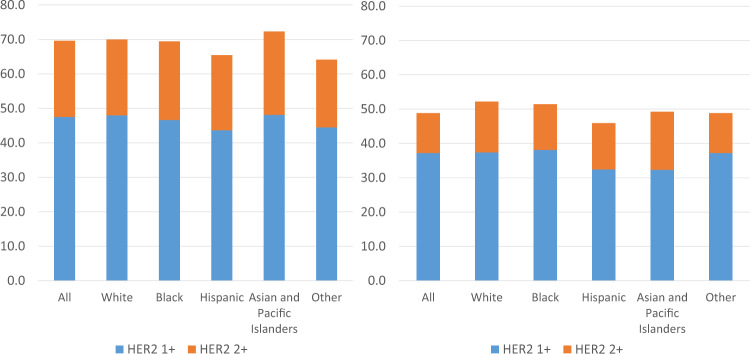
Prevalence of HER2 low tumors by patient race and tumors hormonal receptor status. **A** (Left): Hormonal receptor positive. *P* < 0.001. **B** (Right): Hormonal receptor negative. *P* = 0.27; Chi-Square test was used to compare the prevalence of HER2-low disease across racial/ethinic groups.

30,929 women who met the inclusion criteria were included in the final analysis; 10,293 (33.3%) women had HER2-zero breast cancers, and 20,636 (66.7%) had HER2-low breast cancers. When stratified by hormonal receptor status, 18,066 (69.7%) cases had HER2-low expression among 25,932 HR+/HER2- cases, whereas 2,570 (51.4%) had HER2-low expression among 4997 HR−/HER2− cases. The prevalence of HER2-low tumors was grossly similar across racial groups, with a slightly lower rate in the Hispanic population (Fig. 1). Among all patients with available HER2 information, the median follow-up was 38.2 months (IQR: 25.5 months, 58.2 months). Baseline characteristics compared by HER2 expression status are shown in Table 1.

**Table 1:** Characteristics of patients with HER2-low and HER2-zero metastatic breast cancer.

Variable	Level	*N*	Overall *N* = 30,929	Her2 Low *N* = 20,636	Her2 Zero *N* = 10,293	*P*-value*
Age	18-44 yr	30,929	3366 (10.9%)	2173 (10.5%)	1193 (11.6%)	<0.001
	45–54 yr		5950 (19.2%)	3840 (18.6%)	2110 (20.5%)	
	55–64 yr		8558 (27.7%)	5785 (28.0%)	2773 (26.9%)	
	65–74 yr		7381 (23.9%)	5016 (24.3%)	2365 (23.0%)	
	75 yr+		5674 (18.3%)	3822 (18.5%)	1852 (18.0%)	
Race	1. White	30,929	22,666 (73.3%)	15312 (74.2%)	7354 (71.4%)	<0.001
	2. Black		5177 (16.7%)	3354 (16.3%)	1823 (17.7%)	
	3. Hispanic		1682 (5.4%)	1036 (5.0%)	646 (6.3%)	
	4. Asian and Pacific Islanders		936 (3.0%)	647 (3.1%)	289 (2.8%)	
	5. Other or unknown		468 (1.5%)	287 (1.4%)	181 (1.8%)	
Educational attainment	1. ≥17.6%	30929	6028 (19.5%)	3922 (19.0%)	2106 (20.5%)	<0.001
	2. 10.9–17.5%		7272 (23.5%)	4811 (23.3%)	2461 (23.9%)	
	3. 6.3–10.8%		7924 (25.6%)	5310 (25.7%)	2614 (25.4%)	
	4. <6.3%		6694 (21.6%)	4490 (21.8%)	2204 (21.4%)	
	5. Unknown		3011 (9.7%)	2103 (10.2%)	908 (8.8%)	
Insurance	1. Private	30929	12,774 (41.3%)	8386 (40.6%)	4388 (42.6%)	0.008
	2. Public Insurance		16,348 (52.9%)	11,018 (53.4%)	5330 (51.8%)	
	3. Uninsured		1424 (4.6%)	972 (4.7%)	452 (4.4%)	
	4. Unknown		383 (1.2%)	260 (1.3%)	123 (1.2%)	
Household income	1. <$40,227	30,929	5410 (17.5%)	3522 (17.1%)	1888 (18.3%)	<0.001
	2. $40,227–$50,353		5938 (19.2%)	4004 (19.4%)	1934 (18.8%)	
	3. $50,354–$63,332		6459 (20.9%)	4327 (21.0%)	2132 (20.7%)	
	4. ≥$63,333		10,062 (32.5%)	6648 (32.2%)	3414 (33.2%)	
	5. Unknown		3060 (9.9%)	2135 (10.3%)	925 (9.0%)	
Treatment setting	1. Community Cancer Program	30,929	2129 (6.9%)	1480 (7.2%)	649 (6.3%)	<0.001
	2. Comprehensive Community Cancer Program		11,166 (36.1%)	7655 (37.1%)	3511 (34.1%)	
	3. Academic Comprehensive Cancer Program		10,025 (32.4%)	6372 (30.9%)	3653 (35.5%)	
	4. Integrated Network Cancer Program		5781 (18.7%)	3964 (19.2%)	1817 (17.7%)	
	5. Unknown		1828 (5.9%)	1165 (5.6%)	663 (6.4%)	
Treatment location	1. Metro	30,929	26,079 (84.3%)	17,314 (83.9%)	8765 (85.2%)	0.020
	2. Urban		3536 (11.4%)	2436 (11.8%)	1100 (10.7%)	
	3. Rural		519 (1.7%)	357 (1.7%)	162 (1.6%)	
	4. Unknown		795 (2.6%)	529 (2.6%)	266 (2.6%)	
Histology	1. Ductal adenocarcinoma	30,929	20,394 (65.9%)	13,833 (67.0%)	6561 (63.7%)	<0.001
	2. Lobular adenocarcinoma		4378 (14.2%)	2880 (14.0%)	1498 (14.6%)	
	3. Mixed or unknown histology		6157 (19.9%)	3923 (19.0%)	2234 (21.7%)	
Tumor grade	1. Well differentiated	30,929	2546 (8.2%)	1778 (8.6%)	768 (7.5%)	<0.001
	2. Moderately differentiated		11,985 (38.8%)	8480 (41.1%)	3505 (34.1%)	
	3. Poorly differentiated/Undifferentiated		16,398 (53.0%)	10,378 (50.3%)	6020 (58.5%)	
Visceral metastasis at diagnosis	1. Yes	30,929	13,210 (42.7%)	8724 (42.3%)	4486 (43.6%)	0.029
	2. No		17,719 (57.3%)	11,912 (57.7%)	5807 (56.4%)	
Hormonal receptor status	1. Yes	30,929	25,932 (83.8%)	18,066 (87.5%)	7866 (76.4%)	<0.001
	2. No		4997 (16.2%)	2570 (12.5%)	2427 (23.6%)	
Surgical treatment	1. Lumpectomy or partial mastectomy	30,929	2326 (7.5%)	1506 (7.3%)	820 (8.0%)	0.020
	2. Total mastectomy		5434 (17.6%)	3692 (17.9%)	1742 (16.9%)	
	3. No surgery		23,169 (74.9%)	15,438 (74.8%)	7731 (75.1%)	
Hormone treatment	1. Yes	30,929	22,669 (73.3%)	15,812 (76.6%)	6857 (66.6%)	<0.001
	2. No		8260 (26.7%)	4824 (23.4%)	3436 (33.4%)	
Chemotherapy	1. Yes	30,929	18,428 (59.6%)	11,889 (57.6%)	6539 (63.5%)	<0.001
	2. No		12,501 (40.4%)	8747 (42.4%)	3754 (36.5%)	
Comorbidity score	0	30,929	25,211 (81.5%)	16,891 (81.9%)	8320 (80.8%)	0.014
	1		4052 (13.1%)	2625 (12.7%)	1427 (13.9%)	
	2		1116 (3.6%)	765 (3.7%)	351 (3.4%)	
	> = 3		550 (1.8%)	355 (1.7%)	195 (1.9%)	
Year of diagnosis	2010	30929	2685 (8.7%)	1834 (8.9%)	851 (8.3%)	<0.001
	2011		3059 (9.9%)	2147 (10.4%)	912 (8.9%)	
	2012		3241 (10.5%)	2337 (11.3%)	904 (8.8%)	
	2013		3595 (11.6%)	2413 (11.7%)	1182 (11.5%)	
	2014		4020 (13.0%)	2716 (13.2%)	1304 (12.7%)	
	2015		4216 (13.6%)	2823 (13.7%)	1393 (13.5%)	
	2016		4860 (15.7%)	3129 (15.2%)	1731 (16.8%)	
	2017		5253 (17.0%)	3237 (15.7%)	2016 (19.6%)	

*p-value was calculated using Chi-Square tests.

Women with HER2-low breast cancers were older (relative to women with HER2-zero breast cancers) (75 yr+: 3822/20,636 (18.5%) vs. 1852/10,293 (18.0%); 65-74 yr: 5016/20,636 (24.3%) vs. 2365/10,293 (23.0%), *p* < 0.001), and tended to have public insurance at diagnosis (11,018/20,636 (53.4%) vs. 5330/10,293 (51.8%), *p* = 0.008). In terms of clinical risk factors, HER2-low disease was associated with positive hormonal receptor status (18,066/20,636 (87.5%) vs. 7866/10,293 (76.4%), *p* < 0.001), well or moderately differentiated grade (well-differentiated: 1778/20,636 (8.6%) vs. 768/10.293 (7.5%); moderately differentiated: 8480/20.636 (41.1%) vs. 3505/10.293 (34.1%), *p* < 0.001), ductal histology (13,833/20,636 (67.0%) vs. 6561/10,293 (63.7%), *p* < 0.001), no visceral metastasis at diagnosis (11,912/20,636 (57.7%) vs. 5807/10.293 (56.4%), *p* < 0.001). Furthermore, patients with HER2-low breast cancers were more likely to be diagnosed in the early 2010s (*p* < 0.001), received more hormonal treatment (15,812/20,636 (76.6%) vs. 6857/10,293 (66.6%), *p* < 0.001), but less chemotherapy (11,889/20,636 (57.6%) vs. 6539/10,293 (63.5%), *p* < 0.001) (Table 1).

When stratified by tumor hormonal status, most socio-demographic and clinical factors were well-balanced between HER2-low and HER2-zero tumors. In the HR-positive subgroup, HER2-low tumors were associated with ductal histology, well/moderately differentiated tumors, and diagnosis in the early 2010s. In addition, patients with HR+/HER2-low tumors were slightly more likely to be public insurance beneficiaries and have visceral metastasis at diagnosis than HR+/HER2-zero tumors (Supplementary Table 1A). In the HR-negative subgroup, HER2-low tumors were also associated with well/moderately differentiated tumors, non-visceral metastasis at diagnosis, and a higher probability of diagnosis in the early 2010s (Supplementary Table 1B).

### Kaplan–Meier estimates

**Fig. 2:**
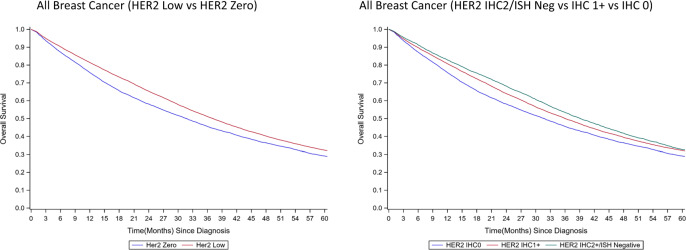
Survival curve by level of HER2 expression. **A** (Left): All breast cancer (HER2 Low vs. HER2 Zero). *P* < 0.001; **B** (Right): All breast cancer (HER2 IHC2/ISH Neg vs. IHC 1+ vs. IHC 0). *P* < 0.001; Log-rank test was used to compare the survival across HER2 expression.

After a median follow-up of 38.2 months, women with HER2-low breast cancer had significantly longer median OS than those with HER2-zero breast cancer (HER2-Low: 37.1 months vs. HER2-Zero: 31.7 months; log-rank *p* < 0.001). In particular, patients with HER2 2+/ISH negative tumors had better OS than those with HER2 1+ and HER2 0 tumors(IHC2+: 39.1 months vs. IHC1+: 36.0 months vs. IHC0: 31.7 months; log-rank *p* < 0.001) (Fig. 2).

**Fig. 3:**
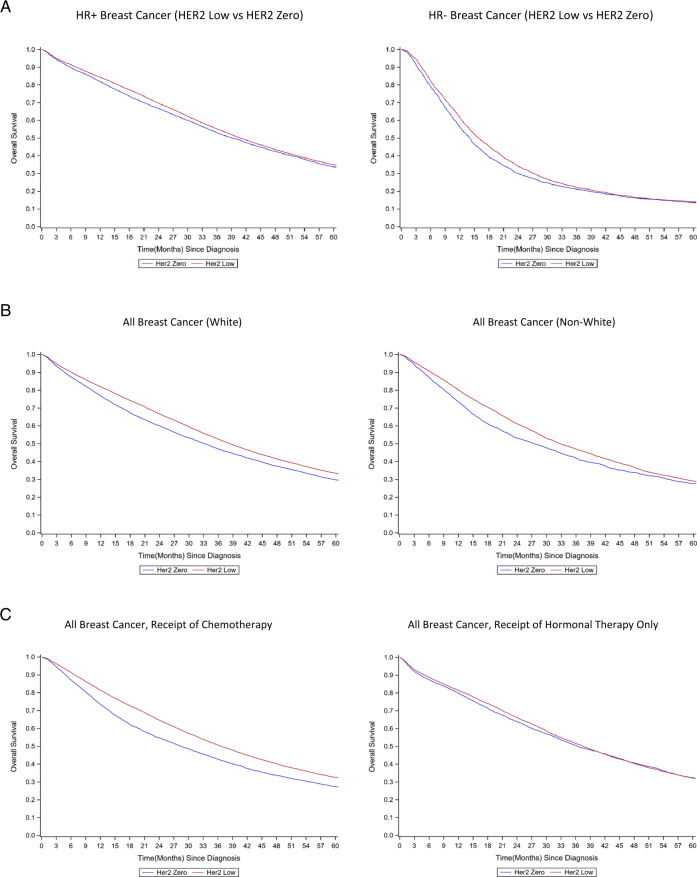
Survival curve by level of HER2 expression and socio-clinical factors. **A** By hormonal receptor status: Left: HR+ breast cancer (HER2 Low vs. HER2 Zero), *p* = 0.003; Right: HR− breast cancer (HER2 Low vs. HER2 Zero), *p* = 0.007. **B** By patients’ race: Left: HR+ breast cancer (White), *p* < 0.001; Right: HR− breast cancer (non-white), *p* < 0.001. **C** By type of treatment: Left: All breast cancer, receipt of chemotherapy, *p* < 0.001; Right all breast cancer, receipt of hormonal therapy only, *p* = 0.43. Log-rank test was used to compare the survival across HER2 expression.

In stratified analyses, similar results were observed in both HR+ and HR− groups (HR-positive: HER2-low: 40.9 months vs. HER2-zero: 39.2 months, log-rank *p* = 0.003, HR-negative: HER2-low: 16.0 months vs. HER2-zero: 14.1 months, log-rank *p* = 0.007) (Fig. 3A, Supplementary Fig. 1a). Both white and non-white patients also shared the survival advantage of HER2-Low tumors (White: HER2-low: 38.4 months vs. HER2-zero: 33.3 months, log-rank *p* < 0.001, Non-white: HER2-low: 32.8 months vs. HER2-zero: 27.5 months, HR 0.88 log-rank *p* < 0.001) (Fig. 3B, Supplementary Fig. 1b). However, the survival advantage of HER2-low tumors was in women who received chemotherapy as the first-line treatment, but not in those who only received hormonal therapy (Chemotherapy: HER2-low: 36.7 months vs. HER2-zero: 28.4 months, log-rank *p* < 0.001, Hormonal therapy only: HER2-low: 37.5 months vs. HER2-zero: 36.5 months, log-rank *p* = 0.43) (Fig. 3C, Supplementary Fig. 1c).

### Multivariable survival analyses

After adjusting for covariates, HER2-low tumors remained associated with prolonged survival (HR 0.95, 95% CI (0.92–0.98), p < 0.001). Similar associations were found in the HR-positive (HR 0.95, 95% CI (0.91–0.98), *p* = 0.002) and HR-negative cohorts (HR 0.92, 95% CI (0.86–0.98), *p* = 0.01). In addition, HER2 2+/ISH negative breast cancer had a small but significant survival advantage compared to HER2 1+ breast cancer (HER2 2+ vs. HER2 1+, HR 0.95, 95% CI (0.92–0.99), *p* = 0.01).

**Fig. 4:**
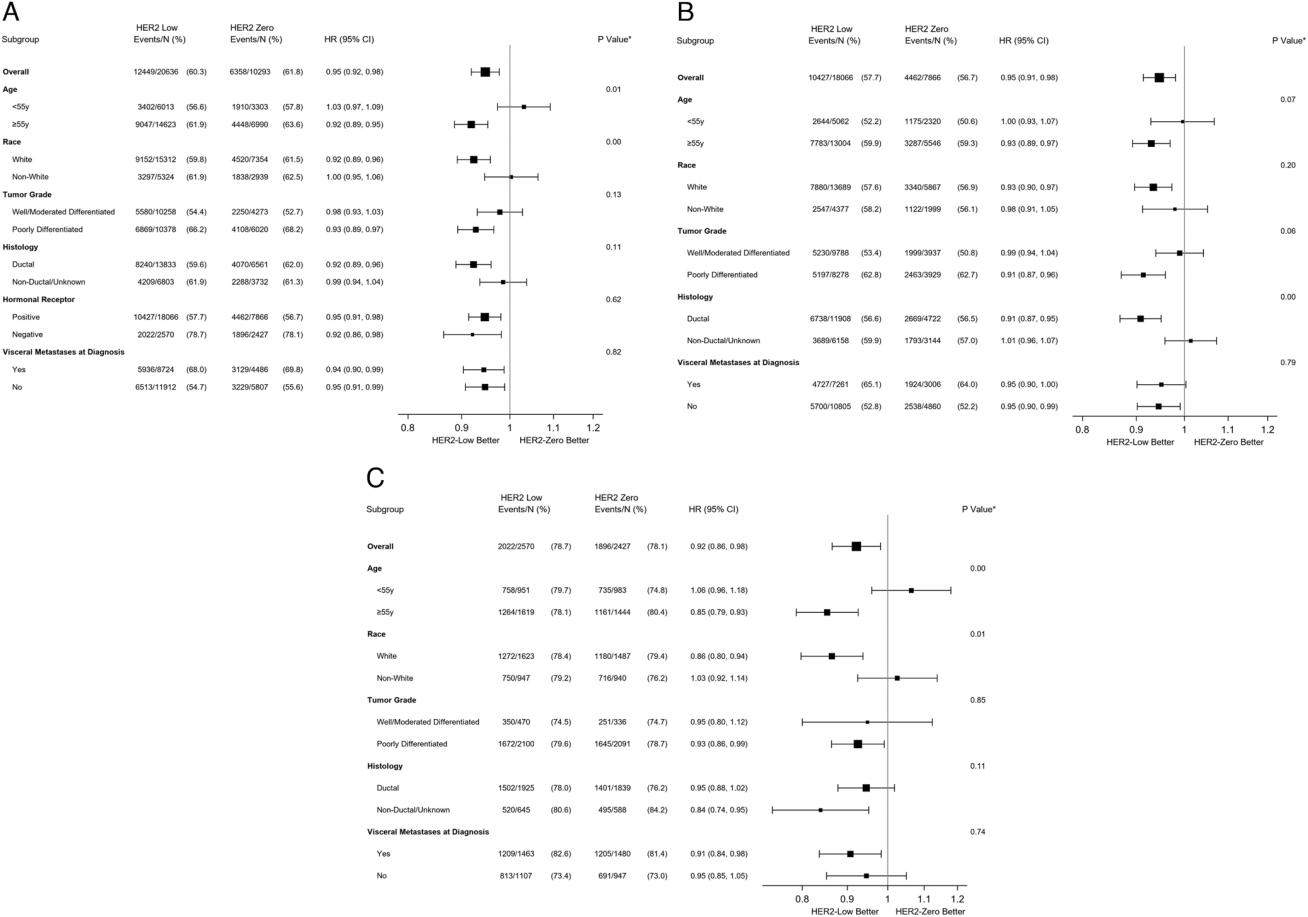
Adjusted hazard ratio (HER2-low vs. HER2-zero breast cancer) from multivariable Cox regression analysis for overall survival. **A** All breast cancer; **B** HR+ breast cancer. **C** HR− Breast cancer. *p*-value is calculated for interaction between HER2 expression level and each subgroup, based on using multivariable cox regression. Error bars showed the confidence interval of the hazard ratio of OS between HER2-low vs. HER2-zero breast cancer. The Cox regression models were adjusted for age, race/ethnicity, household income, comorbidities, location, tumor grade, histology, hormonal receptor status (if applicable), presence of visceral metastasis, the type of cancer center (where women received care), year of diagnosis, and treatment type.

Further subgroup analyses showed the survival advantage of HER2-low tumors was significantly correlated with age (<55 yr: HR 1.03 95% CI (0.97–1.09), vs. ≥55 yr: HR 0.92 95% CI (0.89–0.95), *p*-interaction = 0.01), race (white: HR 0.92 95% CI (0.89–0.96), vs. non-white: HR 1.00 95% CI (0.95–1.06), *p*-interaction < 0.01), receipt of chemotherapy (chemotherapy: HR 0.92 95% CI (0.88–0.96), vs. hormone therapy only: HR 0.99 95% CI (0.94–1.04), *p*-interaction = 0.04). This advantage was not associated with other socio-demographic and clinical factors (all *p*-interaction > 0.05) (Fig. 4A). In the HR-positive population, the survival advantage of HER2-low tumors was more significant among women with ductal adenocarcinoma (*p*-interaction < 0.01), those who received any chemotherapy (*p*-interaction = 0.02) (Fig. 4B). In the HR-negative population, the advantage was more significant among white patients (*p*-interaction = 0.01) and patients aged 55 years or older (*p*-interaction < 0.01) (Fig. 4C)

Sensitivity analyses limited to patients who received diagnosis and treatments at the same reporting facility showed similar results (Supplementary Fig. 2a–c, Supplementary Table 3).

## Discussion

This study found that nearly 70% of HR+/HER2− metastatic breast cancers and 50% of HR−/HER2− metastatic breast cancers had low HER2 expression, with slight variation across racial groups. HER2-low breast cancers had marginally better OS than HER2-zero breast cancers, regardless of their hormone receptor status marginal. The observed survival advantage was primarily observed in white women who received chemotherapy in their first course of treatment.

Understanding the prevalence and clinical outcomes of HER2-low breast cancer may inform the future development of anti-HER2 therapeutics^7^. The interaction between chemotherapy and the survival advantage of HER2-low disease may explain why low HER2 tumors are sensitive to novel antibody–drug conjugates but not traditional anti-HER2 antibodies^5^. Despite our observation, HER2-low tumors had a better outcome than HER2-zero tumors. While the DESTINY-Breast 04 trial changed the clinical practice with a meaningful improvement in OS and PFS for women with pretreated metastatic HR+/HER2-low diseases^12^, researchers need to explore more treatment options in the first-line setting. Our results suggest novel antibody–drug conjugates may have a potential role in the first line especially given the chemosensitivity of these tumors and other studies reporting suboptimal efficacy with CDK 4/6 inhibitors in HER2-low tumors^13^.

Our study is the first comprehensive data to provide epidemiological and outcome data of HER2-low tumors in the black and Hispanic populations. Most previous studies reported white and Chinese populations but included fewer black and Hispanic populations^14–18^. We found a similar prevalence of HER2- low tumors across racial groups in the metastatic setting. In addition, white and non-white patients with HER2-low tumors shared a similar survival advantage in the univariable analysis. However, this survival advantage disappeared in the non-white population with the adjustment of covariates. It suggests that other socio-demographic and clinical factors may fully explain this survival advantage. Another possible explanation for the non-significant results among non-whites is the statistical model overfitting. Future research warrants exploring any biological difference in HER2-low tumors between white and non-white populations. These new findings also suggest the importance of enrollment of non-white women in future clinical trials of HER2-low breast cancer.

Consistent with most previous studies, this study found that tumors with HER2-low expression were more likely to be HR-positive and invasive ductal adenocarcinoma^14,17–19^. In addition, the results showed that HER2-low breast cancers had lower tumor grades and fewer visceral metastases. Our results were partially consistent with a recent US study on HER2-low breast cancer. Tarantino et al. reviewed 5235 stages I–III HER2-negative breast cancer cases operated at Dana-Faber/Brigham Cancer Center and found a clear association of HER2-low expression with low tumor grade and high ER expression, but no significant difference in clinical or pathological parameters^19^. However, it is notable that their study focused mostly on early-stage diseases while our results are based on de novo metastatic diseases. In a Chinese population, Li et al. did not find any difference in proliferation rate (Ki-67 index) and the number of metastatic sites between HER2-low and HER2-zero patients^18^. In another study in HR-positive cancers, HER2-low expression was associated with more expression of luminal-related genes, but less proliferation-related and tyrosine kinase genes. Interestingly, they did not find a similar association in HR-negative breast cancers^17^. In contrast, Dehghani et al. found that HER2 IHC2+/ISH negative tumors had less lymphovascular invasion and fewer basal-like subtypes than HER2-zero tumors in triple-negative breast cancers^14^. Due to the limitation of the NCDB dataset, we could not provide detailed molecular data such as KI-67, or genomic data of the metastatic HER-2 low tumors in this study. Further studies are warranted to explore molecular differences between Her2-low and Her2-zero tumors in the future.

The large sample size of this study allowed us to identify a marginal survival advantage of HER2-low breast cancer in both HR-positive and HR-negative metastatic breast cancer. Further analyses showed that the HER2 IHC score of tumors was correlated with OS. Our results are partially different from previous studies. Li et al. found that HER2-low, especially HER2 IHC2+ tumors, had remarkably better survival than HER2-zero disease in the overall and HR-positive populations, but not in the HR-negative populations^18^. Gampenrieder et al. reported no clear OS difference between HER2-low and HER2-zero tumors in HR+ and HR− breast cancer^16^. Dehghani et al. showed that HER2 2+ breast cancers were associated with better survival than HER2-zero in triple-negative breast cancers.

Tarantino et al. found that HER2-low expression was not associated with survival in patients with early-stage HR+/HER2-negative or HR-/HER2-negative breast cancers^19^. However, a large sample size is essential to identify survival differences in early-stage settings, as these patients tend to have a satisfactory prognosis with a narrow margin in survival. In addition, patients with early-stage and metastatic breast cancers often received different treatments. These conflicting studies were also limited by relatively small size^16^, did not report the impact of treatment on survival of stage IV breast cancer among HER-low and zero breast cancers, or only included about 10% of women with stage IV breast cancers^17,18,20^. The large sample size in our study may provide higher statistical power and more stable estimates of differences in survival between HER2-low and HER2-zero tumors. Meanwhile, the statistical significance may not always translate to clinical significance given the marginal magnitude of difference in OS.

The survival advantage of HER2 low breast cancer seems mostly from chemotherapy in the front line, regardless of tumor HR status. However, we did not observe a similar survival advantage in those who only received hormonal therapy in the first course of treatment. No previous study has examined the interaction between treatment type and survival advantage in HER2-low breast cancer. Considering that low-grade tumor tends to be less sensitive to chemotherapy^21,22^, it is surprising to observe the survival benefit of HER2-low tumors only in patients who received chemotherapy at the front line. Nonetheless, as the NCDB only reports the type of cancer treatment without details for further analyses, future studies are warranted to confirm this interaction in another cohort and explore its potential mechanism.

The major strength of this study is that the NCDB, a national cancer outcome database, fully reflects the diversity of breast cancer patients and the treatments in the US^23^. With a considerably more extensive and more diverse sample, we examined the difference between HER2-low and HER2-zero tumors in most subgroups with adequate statistical power. Another strength of this study is that we examined the interactions between the type of treatment and survival outcomes, which has not been reported previously. Admittedly, our results are only hypothesis-generating due to the observational nature of this study and the limitations of the NCDB dataset. Further studies are warranted to explore this interaction in other real-world outcome datasets with more treatment information.

Admittedly, HER2 low expression is an evolving concept. The ASCO/CAP updated their recommendation on HER2 low expression: included ultralow HER2-expression in IHC 0 group, lowered the IHC2+ cutoff from 30% to 10% stained cells, and eliminated ISH equivocal category during and after the study period (in 2013 and 2018)^24,25^. Our data reflected this change: higher proportion of HER2-zero (a mix of HER2-expressing and non-expressing) tumors were diagnosed in the late 2010s. Notably, such updates may introduce a bias that mildly overestimated the survival difference between IHC2+/1+ and IHC0 tumors but underestimated the real difference between HER2 absence and HER2-low expressing tumors. In fact, many experts argue that IHC may not be the best way to define HER2 expression, as the interrater agreement between HER2 IHC0 and IHC1+ was often suboptimal even in high-quality pathological centers^26^. Additionally, the phase 2 Daisy trial reported a clinically meaningful 30.6% response rate with T-Dxd in IHC 0 cohort^27^. The ongoing DESTINY-Breast06 phase 3 trial in the metastatic setting comparing T-DXd to chemotherapy also enrolls HER2-0 patients (with ultralow expression)^28^. A better diagnostic tool is urgently needed in this space, given that there is a whole spectrum of HER2 expression in HER2-nonamplified tumors, that may potentially benefit from new anti-Her 2 antibody–drug conjugates.

This study has other limitations. The NCDB data is based on hospital data rather than population data. This study should not be interpreted as a nationally representative study despite its large and diverse sample^23^. Due to the nature of cancer registry data in the NCDB, we only captured patients with de novo metastatic breast cancer. In addition, the HER2 IHC and ISH results were based on reports from cancer centers and had not been confirmed in a central pathology laboratory. Moreover, the NCDB recorded biopsy results from one site in most patients and it does not specify where the sample was from (a metastatic site or primary tumor). Thus, the discordance of HER2 status between the primary site and metastatic lesion was not evaluated. In addition, if administrated outside the reporting facility, chemotherapy or hormonal therapy information may be missing. Some women who received treatment outside facilities may be incorrectly classified as having “no chemotherapy” or “no hormonal therapy.” However, the sensitivity analyses confirmed our results even when limiting to the cases with initial diagnosis and all first-course treatment done at the same reporting facility. In addition, the NCDB contains no or limited data on family history, and genetic information such as BRCA1/2. This limits potential correlating analyses of such information with HER-2 status and clinical outcomes. We could not stratify our analysis for the molecular subtype due to the lack of the KI-67 index in the NCDB. Further, the NCDB only recorded the treatment information from the first course, so the exact regimen, numbers of cycles, and treatments beyond the first courses are unknown. Therefore, we cannot adjust specific CDK4/6 inhibitors or checkpoint inhibitors in analyses. However, checkpoint inhibitors were not available on the market during the study period and the majority of patients received the first treatment course before 04/2015 when the US FDA granted accelerated approval to palbociclib, the first CDK 4/6 inhibitor. Lastly, the retrospective nature of this study predisposed the results to other unknown confounders, which we can not adjust with statistical models.

HER2-low tumors have marginally better survival in the metastatic setting than HER2-zero tumors, irrespective of hormone receptor status. The survival advantage was primarily observed in patients who received chemotherapy on the front line. Further research is warranted to confirm this finding and explore its underlying mechanism. More treatment strategies based on novel anti-HER2 therapies, such as trastuzumab deruxtecan, should be sought for metastatic HER-2 low and zero breast cancer.

## Methods

### Patient selections

The NCDB, a joint program from the Commission on Cancer of the American College of Surgeons and the American Cancer Society, is a nationwide oncology outcomes database that collects information on ~70% of all new invasive cancer diagnoses in the United States.

**Fig. 5:**
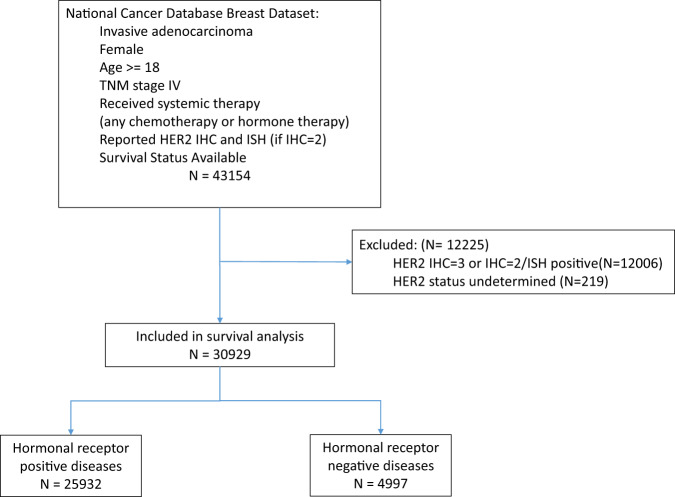
Study design. NCDB National Cancer Database.

Between January 1, 2010, and December 31, 2017, 43,154 women newly diagnosed with breast cancer were identified. Women were included if they had complete information on age, survival status, TNM stage 4 disease at diagnosis, hormonal receptor status (ER+ or PR+), a HER2 expression level (IHC score, and ISH status if IHC2+), and received systemic treatment (including chemotherapy or hormonal therapy). Women were excluded if their tumors were HER2−IHC3+, IHC2+/ISH positive, or undetermined or their survival status was missing (Fig. 5). The treatment coding in the NCDB is limited to the first course of treatment, defined as all treatments noted in the treatment and administrated before disease progression or recurrence.

The HER2 expression level was determined based on HER2 immunohistochemistry (IHC) and in situ hybridization(ISH) summary results (Site-Specific Factor (SSF) 8, SSF11, SSF13)^23^. Both IHC and ISH data were retrieved from the local pathological report without central review. According to the 2018 American Society of Clinical Oncology/College of American Pathologists (ASCO/CAP) guidelines for HER2 testing, HER2 positive was defined as evidence of HER2 overexpression on an IHC assay (score 3+) or gene amplification on an ISH essay of at least one tumor sample. Reflex ISH testing is required to define HER2 status in cases of a 2+ IHC score^4^. To be consistent with previous studies on HER2-low breast cancer, we used the terms HER2-zero (IHC0) and HER2-low (IHC 1+/IHC 2+ with negative ISH) in this study. Overall survival (OS) was the primary endpoint.

### Statistical analysis

Data were analyzed from October 1, 2021, through March 1, 2022. Baseline characteristics between HER2-low and HER2-zero groups were compared using the Chi-square test. Similarly, baseline characteristics were also compared across HER2 expression levels (1+ vs 2+) using the Chi-square test. Survival curves for OS were estimated using the Kaplan–Meier method, and a log-rank test was used to compare OS across HER2 expression levels.

Three multivariable Cox regression models were used for all, HR-positive and HR-negative cohorts to estimate hazard ratios (HR), adjusting for socio-demographic and clinical covariates, including age, race/ethnicity, household income, comorbidities, location, tumor grade, histology, presence of visceral metastasis, the type of cancer center (where women received care), year of diagnosis, and treatment type. In the exploratory subgroup analyses of all three cohorts, we examined the interaction between HER2 expression level and each of the important socio-demographic and clinical factors in separate multivariable cox regression models, adjusting for the same covariates.

To minimize misclassification bias from different reporting facilities, we performed the sensitivity analyses limiting to the cases with initial diagnosis and all first-course treatment given at the same reporting facility.

All statistical analyses were performed using SAS software, version 9.4 (SAS Institute Inc.). All statistical significance testing was two-sided at *p* < 0.05.

The NCDB data were deidentified and compliant with HIPAA. Patient informed consent is not obtained prior to institutional data submission to NCDB. As the study used publicly available data upon request, with no attempt made to contact or re-identify the subject, institutional review board review was exempted by the Office of Research Subject Protection at the Roswell Park Cancer Comprehensive Cancer Center.

## Supplementary information

SUPPLEMENTAL MATERIAL

Supplementary Figures

## Data Availability

The primary dataset (National Cancer Database) is available publicly through the American College of Surgeons (https://www.facs.org/quality-programs/cancer/ncdb), but restrictions apply to the availability of these data, which were used under license for the current study and so are not publicly available. Data are however available from the authors upon reasonable request and with permission of the NCDB.

## Code availability

Analysis code is available from the corresponding author upon reasonable request.
