# Supplementary material for: Clinical outcomes of de novo metastatic HER2-low breast cancer: a National Cancer Database Analysis
Source: NPJ Breast Cancer. 2022 Dec 30;8:135. doi: 10.1038/s41523-022-00498-8 (PMC9803673; doi:10.1038/s41523-022-00498-8)
Supplement: Supplementary file 1 — SUPPLEMENTAL MATERIAL [file 41523_2022_498_MOESM1_ESM.pdf]

Supplementary Table1A. Characteristics of Patients with HER2-Low and HER2-Zero, HR+ Metastatic Breast Cancer.

**Variable**	**Level**	**N**	**Overall N=25932**	**Her2 Low N=18066**	**Her2 Zero N=7866**	**P-Value***
Age	18-44y	25932	2616 (10.1%)	1820 (10.1%)	796 (10.1%)	**0.022**
	45-54y		4766 (18.4%)	3242 (17.9%)	1524 (19.4%)	
	55-64y		7139 (27.5%)	5048 (27.9%)	2091 (26.6%)	
	65-74y		6342 (24.5%)	4453 (24.6%)	1889 (24.0%)	
	75y+		5069 (19.5%)	3503 (19.4%)	1566 (19.9%)	
Race	1. White	25932	19556 (75.4%)	13689 (75.8%)	5867 (74.6%)	**<.001**
	2. Black		3839 (14.8%)	2666 (14.8%)	1173 (14.9%)	
	3. Hispanic		1349 (5.2%)	883 (4.9%)	466 (5.9%)	
	4. Asian and Pacific Islanders		806 (3.1%)	583 (3.2%)	223 (2.8%)	
	5. Other or unknown		382 (1.5%)	245 (1.4%)	137 (1.7%)	
Educational Attainment	1. >=17.6%	25932	4850 (18.7%)	3348 (18.5%)	1502 (19.1%)	0.061
	2. 10.9% - 17.5%		5997 (23.1%)	4168 (23.1%)	1829 (23.3%)	
	3. 6.3% - 10.8%		6693 (25.8%)	4640 (25.7%)	2053 (26.1%)	
	4. <6.3%		5761 (22.2%)	4012 (22.2%)	1749 (22.2%)	
	5. Unknown		2631 (10.1%)	1898 (10.5%)	733 (9.3%)	
Insurance	1. Private	25932	10594 (40.9%)	7293 (40.4%)	3301 (42.0%)	**0.018**
	2. Public Insurance		13900 (53.6%)	9731 (53.9%)	4169 (53.0%)	
	3. Uninsured		1123 (4.3%)	819 (4.5%)	304 (3.9%)	
	4. Unknown		315 (1.2%)	223 (1.2%)	92 (1.2%)	
Household Income	1. <$40,227	25932	4291 (16.5%)	2967 (16.4%)	1324 (16.8%)	**<.001**
	2. $40,227 - $50,353		4872 (18.8%)	3465 (19.2%)	1407 (17.9%)	
	3. $50,354 - $63,332		5477 (21.1%)	3797 (21.0%)	1680 (21.4%)	
	4. >=$63,333		8626 (33.3%)	5912 (32.7%)	2714 (34.5%)	
	5. Unknown		2666 (10.3%)	1925 (10.7%)	741 (9.4%)	

Supplementary Table1A. Characteristics of Patients with HER2-Low and HER2-Zero, HR+ Metastatic Breast Cancer.

Variable	Level	N	Overall N=25932	Her2 Low N=18066	Her2 Zero N=7866	P-Value*
Treatment Setting	1. Community Cancer Program	25932	1820 (7.0%)	1311 (7.3%)	509 (6.5%)	**<.001**
	2. Comprehensive Community Cancer Program		9454 (36.5%)	6742 (37.3%)	2712 (34.5%)	
	3. Academic Comprehensive Cancer Program		8366 (32.3%)	5554 (30.7%)	2812 (35.7%)	
	4. Integrated Network Cancer Program		4871 (18.8%)	3466 (19.2%)	1405 (17.9%)	
	5. Unknown		1421 (5.5%)	993 (5.5%)	428 (5.4%)	
Treatment Location	1. Metro	25932	21871 (84.3%)	15158 (83.9%)	6713 (85.3%)	**0.004**
	2. Urban		2944 (11.4%)	2137 (11.8%)	807 (10.3%)	
	3. Rural		434 (1.7%)	302 (1.7%)	132 (1.7%)	
	4. Unknown		683 (2.6%)	469 (2.6%)	214 (2.7%)	
Histology	1. Ductal adenocarcinoma	25932	16630 (64.1%)	11908 (65.9%)	4722 (60.0%)	**<.001**
	2. Lobular adenocarcinoma		4216 (16.3%)	2787 (15.4%)	1429 (18.2%)	
	3. Mixed or unknown histology		5086 (19.6%)	3371 (18.7%)	1715 (21.8%)	
Tumor Grade	1. Well differentiated	25932	2501 (9.6%)	1750 (9.7%)	751 (9.5%)	**<.001**
	2. Moderately differentiated		11224 (43.3%)	8038 (44.5%)	3186 (40.5%)	
	3. Poorly differentiated/Undifferentiated		12207 (47.1%)	8278 (45.8%)	3929 (49.9%)	
Visceral Metastasis at Diagnosis	1. Yes	25932	10267 (39.6%)	7261 (40.2%)	3006 (38.2%)	**0.003**
	2. No		15665 (60.4%)	10805 (59.8%)	4860 (61.8%)	
Hormonal Receptor Status	1. Yes	25932	25932 (100.0%)	18066 (100.0%)	7866 (100.0%)	**-**
Surgical Treatment	1. Lumpectomy or partial mastectomy	25932	1910 (7.4%)	1315 (7.3%)	595 (7.6%)	**0.001**
	2. Total mastectomy		4267 (16.5%)	3074 (17.0%)	1193 (15.2%)	
	3. No surgery		19755 (76.2%)	13677 (75.7%)	6078 (77.3%)	

Supplementary Table1A. Characteristics of Patients with HER2-Low and HER2-Zero, HR+ Metastatic Breast Cancer.

Variable	Level	N	Overall N=25932	Her2 Low N=18066	Her2 Zero N=7866	P-Value*
Hormone Treatment	1. Yes	25932	22445 (86.6%)	15689 (86.8%)	6756 (85.9%)	**0.038**
	2. No		3487 (13.4%)	2377 (13.2%)	1110 (14.1%)	
Chemotherapy	1. Yes	25932	13509 (52.1%)	9364 (51.8%)	4145 (52.7%)	0.201
	2. No		12423 (47.9%)	8702 (48.2%)	3721 (47.3%)	
Comorbidity Score	0	25932	21133 (81.5%)	14774 (81.8%)	6359 (80.8%)	0.062
	1		3373 (13.0%)	2292 (12.7%)	1081 (13.7%)	
	2		955 (3.7%)	681 (3.8%)	274 (3.5%)	
	>=3		471 (1.8%)	319 (1.8%)	152 (1.9%)	
Year of Diagnosis	2010	25932	2223 (8.6%)	1591 (8.8%)	632 (8.0%)	**<.001**
	2011		2542 (9.8%)	1845 (10.2%)	697 (8.9%)	
	2012		2748 (10.6%)	2058 (11.4%)	690 (8.8%)	
	2013		3035 (11.7%)	2111 (11.7%)	924 (11.7%)	
	2014		3356 (12.9%)	2387 (13.2%)	969 (12.3%)	
	2015		3486 (13.4%)	2442 (13.5%)	1044 (13.3%)	
	2016		4112 (15.9%)	2778 (15.4%)	1334 (17.0%)	
	2017		4430 (17.1%)	2854 (15.8%)	1576 (20.0%)	

*p-value was calculated using Chi-Square tests.

Supplementary Table1B. Characteristics of Patients with HER2-Low and HER2-Zero, HR- Metastatic Breast Cancer.

Variable	Level	N	Overall N=4997	Her2 Low N=2570	Her2 Zero N=2427	P-Value*
Age	18-44y	4997	750 (15.0%)	353 (13.7%)	397 (16.4%)	**0.043**
	45-54y		1184 (23.7%)	598 (23.3%)	586 (24.1%)	
	55-64y		1419 (28.4%)	737 (28.7%)	682 (28.1%)	
	65-74y		1039 (20.8%)	563 (21.9%)	476 (19.6%)	
	75y+		605 (12.1%)	319 (12.4%)	286 (11.8%)	
Race	1. White	4997	3110 (62.2%)	1623 (63.2%)	1487 (61.3%)	0.267
	2. Black		1338 (26.8%)	688 (26.8%)	650 (26.8%)	
	3. Hispanic		333 (6.7%)	153 (6.0%)	180 (7.4%)	
	4. Asian and Pacific Islanders		130 (2.6%)	64 (2.5%)	66 (2.7%)	
	5. Other or unknown		86 (1.7%)	42 (1.6%)	44 (1.8%)	
Educational Attainment	1. >=17.6%	4997	1178 (23.6%)	574 (22.3%)	604 (24.9%)	0.053
	2. 10.9% - 17.5%		1275 (25.5%)	643 (25.0%)	632 (26.0%)	
	3. 6.3% - 10.8%		1231 (24.6%)	670 (26.1%)	561 (23.1%)	
	4. <6.3%		933 (18.7%)	478 (18.6%)	455 (18.7%)	
	5. Unknown		380 (7.6%)	205 (8.0%)	175 (7.2%)	
Insurance	1. Private	4997	2180 (43.6%)	1093 (42.5%)	1087 (44.8%)	0.388
	2. Public Insurance		2448 (49.0%)	1287 (50.1%)	1161 (47.8%)	
	3. Uninsured		301 (6.0%)	153 (6.0%)	148 (6.1%)	
	4. Unknown		68 (1.4%)	37 (1.4%)	31 (1.3%)	
Household Income	1. <$40,227	4997	1119 (22.4%)	555 (21.6%)	564 (23.2%)	0.294
	2. $40,227 - $50,353		1066 (21.3%)	539 (21.0%)	527 (21.7%)	
	3. $50,354 - $63,332		982 (19.7%)	530 (20.6%)	452 (18.6%)	
	4. >=$63,333		1436 (28.7%)	736 (28.6%)	700 (28.8%)	
	5. Unknown		394 (7.9%)	210 (8.2%)	184 (7.6%)	

Supplementary Table1B. Characteristics of Patients with HER2-Low and HER2-Zero, HR- Metastatic Breast Cancer.

Variable	Level	N	Overall N=4997	Her2 Low N=2570	Her2 Zero N=2427	P-Value*
Treatment Setting	1. Community Cancer Program	4997	309 (6.2%)	169 (6.6%)	140 (5.8%)	**<.001**
	2. Comprehensive Community Cancer Program		1712 (34.3%)	913 (35.5%)	799 (32.9%)	
	3. Academic Comprehensive Cancer Program		1659 (33.2%)	818 (31.8%)	841 (34.7%)	
	4. Integrated Network Cancer Program		910 (18.2%)	498 (19.4%)	412 (17.0%)	
	5. Unknown		407 (8.1%)	172 (6.7%)	235 (9.7%)	
Treatment Location	1. Metro	4997	4208 (84.2%)	2156 (83.9%)	2052 (84.5%)	0.091
	2. Urban		592 (11.8%)	299 (11.6%)	293 (12.1%)	
	3. Rural		85 (1.7%)	55 (2.1%)	30 (1.2%)	
	4. Unknown		112 (2.2%)	60 (2.3%)	52 (2.1%)	
Histology	1. Ductal adenocarcinoma	4997	3764 (75.3%)	1925 (74.9%)	1839 (75.8%)	0.294
	2. Lobular adenocarcinoma		162 (3.2%)	93 (3.6%)	69 (2.8%)	
	3. Mixed or unknown histology		1071 (21.4%)	552 (21.5%)	519 (21.4%)	
Tumor Grade	1. Well differentiated	4997	45 (0.9%)	28 (1.1%)	17 (0.7%)	**<.001**
	2. Moderately differentiated		761 (15.2%)	442 (17.2%)	319 (13.1%)	
	3. Poorly differentiated/Undiff erentiated		4191 (83.9%)	2100 (81.7%)	2091 (86.2%)	
Visceral Metastasis at Diagnosis	1. Yes	4997	2943 (58.9%)	1463 (56.9%)	1480 (61.0%)	**0.004**
	2. No		2054 (41.1%)	1107 (43.1%)	947 (39.0%)	
Hormonal Receptor Status	2. No	4997	4997 (100.0%)	2570 (100.0%)	2427 (100.0%)	-
Surgical Treatment	1. Lumpectomy or partial mastectomy	4997	416 (8.3%)	191 (7.4%)	225 (9.3%)	**0.045**
	2. Total mastectomy		1167 (23.4%)	618 (24.0%)	549 (22.6%)	
	3. No surgery		3414 (68.3%)	1761 (68.5%)	1653 (68.1%)	

Supplementary Table1B. Characteristics of Patients with HER2-Low and HER2-Zero, HR- Metastatic Breast Cancer.

Variable	Level	N	Overall N=4997	Her2 Low N=2570	Her2 Zero N=2427	P-Value*
Comorbidity Score	0	4997	4078 (81.6%)	2117 (82.4%)	1961 (80.8%)	0.384
	1		679 (13.6%)	333 (13.0%)	346 (14.3%)	
	2		161 (3.2%)	84 (3.3%)	77 (3.2%)	
	>=3		79 (1.6%)	36 (1.4%)	43 (1.8%)	
Year of Diagnosis	2010	4997	462 (9.2%)	243 (9.5%)	219 (9.0%)	**<.001**
	2011		517 (10.3%)	302 (11.8%)	215 (8.9%)	
	2012		493 (9.9%)	279 (10.9%)	214 (8.8%)	
	2013		560 (11.2%)	302 (11.8%)	258 (10.6%)	
	2014		664 (13.3%)	329 (12.8%)	335 (13.8%)	
	2015		730 (14.6%)	381 (14.8%)	349 (14.4%)	
	2016		748 (15.0%)	351 (13.7%)	397 (16.4%)	
	2017		823 (16.5%)	383 (14.9%)	440 (18.1%)	

*p-value was calculated using Chi-Square tests.

Supplementary Table2A. Characteristics of Patients with HER2 2+ and HER2 1+, HR+ Metastatic Breast Cancer.

Variable	Level	N	Her2 2+ N=5745	Her2 1+ N=12321	P-Value*
Age	18-44y	18066	606 (10.5%)	1214 (9.9%)	0.548
	45-54y		1035 (18.0%)	2207 (17.9%)	
	55-64y		1591 (27.7%)	3457 (28.1%)	
	65-74y		1425 (24.8%)	3028 (24.6%)	
	75y+		1088 (18.9%)	2415 (19.6%)	
Race	1. White	18066	4306 (75.0%)	9383 (76.2%)	0.430
	2. Black		875 (15.2%)	1791 (14.5%)	
	3. Hispanic		294 (5.1%)	589 (4.8%)	
	4. Asian and Pacific Islanders		195 (3.4%)	388 (3.1%)	
	5. Other or unknown		75 (1.3%)	170 (1.4%)	
Educational Attainment	1. >=17.6%	18066	1088 (18.9%)	2260 (18.3%)	**0.021**
	2. 10.9% - 17.5%		1344 (23.4%)	2824 (22.9%)	
	3. 6.3% - 10.8%		1384 (24.1%)	3256 (26.4%)	
	4. <6.3%		1303 (22.7%)	2709 (22.0%)	
	5. Unknown		626 (10.9%)	1272 (10.3%)	
Insurance	1. Private	18066	2280 (39.7%)	5013 (40.7%)	0.166
	2. Public Insurance		3118 (54.3%)	6613 (53.7%)	
	3. Uninsured		283 (4.9%)	536 (4.4%)	
	4. Unknown		64 (1.1%)	159 (1.3%)	
Household Income	1. <$40,227	18066	991 (17.2%)	1976 (16.0%)	0.191
	2. $40,227 - $50,353		1071 (18.6%)	2394 (19.4%)	
	3. $50,354 - $63,332		1192 (20.7%)	2605 (21.1%)	
	4. >=$63,333		1861 (32.4%)	4051 (32.9%)	
	5. Unknown		630 (11.0%)	1295 (10.5%)	

Supplementary Table2A. Characteristics of Patients with HER2 2+ and HER2 1+, HR+ Metastatic Breast Cancer.

Variable	Level	N	Her2 2+ N=5745	Her2 1+ N=12321	P-Value*
Treatment Setting	1. Community Cancer Program	18066	393 (6.8%)	918 (7.5%)	0.167
	2. Comprehensive Community Cancer Program		2136 (37.2%)	4606 (37.4%)	
	3. Academic Comprehensive Cancer Program		1812 (31.5%)	3742 (30.4%)	
	4. Integrated Network Cancer Program		1071 (18.6%)	2395 (19.4%)	
	5. Unknown		333 (5.8%)	660 (5.4%)	
Treatment Location	1. Metro	18066	4816 (83.8%)	10342 (83.9%)	0.361
	2. Urban		671 (11.7%)	1466 (11.9%)	
	3. Rural		92 (1.6%)	210 (1.7%)	
	4. Unknown		166 (2.9%)	303 (2.5%)	
Histology	1. Ductal adenocarcinoma	18066	3955 (68.8%)	7953 (64.5%)	**<.001**
	2. Lobular adenocarcinoma		798 (13.9%)	1989 (16.1%)	
	3. Mixed or unknown histology		992 (17.3%)	2379 (19.3%)	
Tumor Grade	1. Well differentiated	18066	513 (8.9%)	1237 (10.0%)	**0.005**
	2. Moderately differentiated		2644 (46.0%)	5394 (43.8%)	
	3. Poorly differentiated/Undifferentiated		2588 (45.0%)	5690 (46.2%)	
Visceral Metastasis at Diagnosis	1. Yes	18066	2367 (41.2%)	4894 (39.7%)	0.059
	2. No		3378 (58.8%)	7427 (60.3%)	
Hormonal Receptor Status	1. Yes	18066	5745 (100.0%)	12321 (100.0%)	-
Surgical Treatment	1. Lumpectomy or partial mastectomy	18066	412 (7.2%)	903 (7.3%)	0.062
	2. Total mastectomy		1033 (18.0%)	2041 (16.6%)	
	3. No surgery		4300 (74.8%)	9377 (76.1%)	

Supplementary Table2A. Characteristics of Patients with HER2 2+ and HER2 1+, HR+ Metastatic Breast Cancer.

Variable	Level	N	Her2 2+ N=5745	Her2 1+ N=12321	P-Value*
Chemotherapy	1. Yes	18066	3005 (52.3%)	6359 (51.6%)	0.384
	2. No		2740 (47.7%)	5962 (48.4%)	
Comorbidity Score	0	18066	4755 (82.8%)	10019 (81.3%)	**0.046**
	1		703 (12.2%)	1589 (12.9%)	
	2		203 (3.5%)	478 (3.9%)	
	>=3		84 (1.5%)	235 (1.9%)	
Year of Diagnosis	2010	18066	485 (8.4%)	1106 (9.0%)	**0.019**
	2011		599 (10.4%)	1246 (10.1%)	
	2012		729 (12.7%)	1329 (10.8%)	
	2013		657 (11.4%)	1454 (11.8%)	
	2014		748 (13.0%)	1639 (13.3%)	
	2015		775 (13.5%)	1667 (13.5%)	
	2016		879 (15.3%)	1899 (15.4%)	
	2017		873 (15.2%)	1981 (16.1%)	

*p-value was calculated using Chi-Square tests.

Supplementary Table2A. Characteristics of Patients with HER2 2+ and HER2 1+, HR- Metastatic Breast Cancer.

Variable	Level	N	Her2 2+ N=715	Her2 1+ N=1855	P-Value*
Age	18-44y	2570	65 (9.1%)	288 (15.5%)	**<.001**
	45-54y		165 (23.1%)	433 (23.3%)	
	55-64y		212 (29.7%)	525 (28.3%)	
	65-74y		180 (25.2%)	383 (20.6%)	
	75y+		93 (13.0%)	226 (12.2%)	
Race	1. White	2570	460 (64.3%)	1163 (62.7%)	0.490
	2. Black		178 (24.9%)	510 (27.5%)	
	3. Hispanic		45 (6.3%)	108 (5.8%)	
	4. Asian and Pacific Islanders		22 (3.1%)	42 (2.3%)	
	5. Other or unknown		10 (1.4%)	32 (1.7%)	
Educational Attainment	1. >=17.6%	2570	154 (21.5%)	420 (22.6%)	0.920
	2. 10.9% - 17.5%		181 (25.3%)	462 (24.9%)	
	3. 6.3% - 10.8%		185 (25.9%)	485 (26.1%)	
	4. <6.3%		133 (18.6%)	345 (18.6%)	
	5. Unknown		62 (8.7%)	143 (7.7%)	
Insurance	1. Private	2570	280 (39.2%)	813 (43.8%)	0.079
	2. Public Insurance		384 (53.7%)	903 (48.7%)	
	3. Uninsured		44 (6.2%)	109 (5.9%)	
	4. Unknown		7 (1.0%)	30 (1.6%)	
Household Income	1. <$40,227	2570	146 (20.4%)	409 (22.0%)	0.600
	2. $40,227 - $50,353		162 (22.7%)	377 (20.3%)	
	3. $50,354 - $63,332		145 (20.3%)	385 (20.8%)	
	4. >=$63,333		199 (27.8%)	537 (28.9%)	
	5. Unknown		63 (8.8%)	147 (7.9%)	

Supplementary Table2A. Characteristics of Patients with HER2 2+ and HER2 1+, HR- Metastatic Breast Cancer.

Variable	Level	N	Her2 2+ N=715	Her2 1+ N=1855	P-Value*
Treatment Setting	1. Community Cancer Program	2570	47 (6.6%)	122 (6.6%)	**0.013**
	2. Comprehensive Community Cancer Program		249 (34.8%)	664 (35.8%)	
	3. Academic Comprehensive Cancer Program		243 (34.0%)	575 (31.0%)	
	4. Integrated Network Cancer Program		147 (20.6%)	351 (18.9%)	
	5. Unknown		29 (4.1%)	143 (7.7%)	
Treatment Location	1. Metro	2570	605 (84.6%)	1551 (83.6%)	0.247
	2. Urban		73 (10.2%)	226 (12.2%)	
	3. Rural		15 (2.1%)	40 (2.2%)	
	4. Unknown		22 (3.1%)	38 (2.0%)	
Histology	1. Ductal adenocarcinoma	2570	532 (74.4%)	1393 (75.1%)	0.926
	2. Lobular adenocarcinoma		27 (3.8%)	66 (3.6%)	
	3. Mixed or unknown histology		156 (21.8%)	396 (21.3%)	
Tumor Grade	1. Well differentiated	2570	4 (0.6%)	24 (1.3%)	0.097
	2. Moderately differentiated		136 (19.0%)	306 (16.5%)	
	3. Poorly differentiated/Undifferentiated		575 (80.4%)	1525 (82.2%)	
Visceral Metastasis at Diagnosis	1. Yes	2570	403 (56.4%)	1060 (57.1%)	0.721
	2. No		312 (43.6%)	795 (42.9%)	
Surgical Treatment	1. Lumpectomy or partial mastectomy	2570	45 (6.3%)	146 (7.9%)	0.343
	2. Total mastectomy		169 (23.6%)	449 (24.2%)	
	3. No surgery		501 (70.1%)	1260 (67.9%)	

Supplementary Table2A. Characteristics of Patients with HER2 2+ and HER2 1+, HR- Metastatic Breast Cancer.

Variable	Level	N	Her2 2+ N=715	Her2 1+ N=1855	P-Value*
Comorbidity Score	0	2570	586 (82.0%)	1531 (82.5%)	0.526
	1		92 (12.9%)	241 (13.0%)	
	2		23 (3.2%)	61 (3.3%)	
	>=3		14 (2.0%)	22 (1.2%)	
Year of Diagnosis	2010	2570	52 (7.3%)	191 (10.3%)	**0.011**
	2011		86 (12.0%)	216 (11.6%)	
	2012		73 (10.2%)	206 (11.1%)	
	2013		80 (11.2%)	222 (12.0%)	
	2014		85 (11.9%)	244 (13.2%)	
	2015		97 (13.6%)	284 (15.3%)	
	2016		109 (15.2%)	242 (13.0%)	
	2017		133 (18.6%)	250 (13.5%)	

*p-value was calculated using Chi-Square tests.

Supplementary Table3. Characteristics of Patients with HER2-Low and HER2-Zero Metastatic Breast Cancer (Sensitivity analysis in patients whose initial diagnosis and first course treatment given at the same facility.)

Variable	Level	N	Overall N=15556	Her2 Low N=10448	Her2 Zero N=5108	P-Value*
Age	18-44y	15556	1464 (9.4%)	966 (9.2%)	498 (9.7%)	**0.001**
	45-54y		2801 (18.0%)	1806 (17.3%)	995 (19.5%)	
	55-64y		4333 (27.9%)	2933 (28.1%)	1400 (27.4%)	
	65-74y		3796 (24.4%)	2627 (25.1%)	1169 (22.9%)	
	75y+		3162 (20.3%)	2116 (20.3%)	1046 (20.5%)	
Race	1. White	15556	11267 (72.4%)	7628 (73.0%)	3639 (71.2%)	**0.022**
	2. Black		2827 (18.2%)	1868 (17.9%)	959 (18.8%)	
	3. Hispanic		781 (5.0%)	493 (4.7%)	288 (5.6%)	
	4. Asian and Pacific Islanders		473 (3.0%)	328 (3.1%)	145 (2.8%)	
	5. Other or unknown		208 (1.3%)	131 (1.3%)	77 (1.5%)	
Educational Attainment	1. >=17.6%	15556	3188 (20.5%)	2078 (19.9%)	1110 (21.7%)	**0.012**
	2. 10.9% - 17.5%		3715 (23.9%)	2504 (24.0%)	1211 (23.7%)	
	3. 6.3% - 10.8%		3930 (25.3%)	2637 (25.2%)	1293 (25.3%)	
	4. <6.3%		3367 (21.6%)	2273 (21.8%)	1094 (21.4%)	
	5. Unknown		1356 (8.7%)	956 (9.2%)	400 (7.8%)	
Insurance	1. Private	15556	5911 (38.0%)	3895 (37.3%)	2016 (39.5%)	**0.010**
	2. Public Insurance		8640 (55.5%)	5841 (55.9%)	2799 (54.8%)	
	3. Uninsured		827 (5.3%)	586 (5.6%)	241 (4.7%)	
	4. Unknown		178 (1.1%)	126 (1.2%)	52 (1.0%)	
Household Income	1. <$40,227	15556	2948 (19.0%)	1960 (18.8%)	988 (19.3%)	0.073
	2. $40,227 - $50,353		2961 (19.0%)	2008 (19.2%)	953 (18.7%)	
	3. $50,354 - $63,332		3287 (21.1%)	2194 (21.0%)	1093 (21.4%)	
	4. >=$63,333		4984 (32.0%)	3318 (31.8%)	1666 (32.6%)	
	5. Unknown		1376 (8.8%)	968 (9.3%)	408 (8.0%)	

Supplementary Table3. Characteristics of Patients with HER2-Low and HER2-Zero Metastatic Breast Cancer (Sensitivity analysis in patients whose initial diagnosis and first course treatment given at the same facility.)

Variable	Level	N	Overall N=15556	Her2 Low N=10448	Her2 Zero N=5108	P-Value*
Treatment Setting	1. Community Cancer Program	15556	1234 (7.9%)	864 (8.3%)	370 (7.2%)	**<.001**
	2. Comprehensive Community Cancer Program		6046 (38.9%)	4160 (39.8%)	1886 (36.9%)	
	3. Academic Comprehensive Cancer Program		4563 (29.3%)	2869 (27.5%)	1694 (33.2%)	
	4. Integrated Network Cancer Program		2934 (18.9%)	2046 (19.6%)	888 (17.4%)	
	5. Unknown		779 (5.0%)	509 (4.9%)	270 (5.3%)	
Treatment Location	1. Metro	15556	13368 (85.9%)	8938 (85.5%)	4430 (86.7%)	0.120
	2. Urban		1671 (10.7%)	1166 (11.2%)	505 (9.9%)	
	3. Rural		247 (1.6%)	164 (1.6%)	83 (1.6%)	
	4. Unknown		270 (1.7%)	180 (1.7%)	90 (1.8%)	
Histology	1. Ductal adenocarcinoma	15556	10173 (65.4%)	6966 (66.7%)	3207 (62.8%)	**<.001**
	2. Lobular adenocarcinoma		2180 (14.0%)	1442 (13.8%)	738 (14.4%)	
	3. Mixed or unknown histology		3203 (20.6%)	2040 (19.5%)	1163 (22.8%)	
Tumor Grade	1. Well differentiated	15556	1316 (8.5%)	928 (8.9%)	388 (7.6%)	**<.001**
	2. Moderately differentiated		5907 (38.0%)	4208 (40.3%)	1699 (33.3%)	
	3. Poorly differentiated/Undifferentiated		8333 (53.6%)	5312 (50.8%)	3021 (59.1%)	
Visceral Metastasis at Diagnosis	1. Yes	15556	6809 (43.8%)	4501 (43.1%)	2308 (45.2%)	**0.013**
	2. No		8747 (56.2%)	5947 (56.9%)	2800 (54.8%)	
Hormonal Receptor Status	1. Yes	15556	13161 (84.6%)	9191 (88.0%)	3970 (77.7%)	**<.001**
	2. No		2395 (15.4%)	1257 (12.0%)	1138 (22.3%)	

Supplementary Table3. Characteristics of Patients with HER2-Low and HER2-Zero Metastatic Breast Cancer (Sensitivity analysis in patients whose initial diagnosis and first course treatment given at the same facility.)

Variable	Level	N	Overall N=15556	Her2 Low N=10448	Her2 Zero N=5108	P-Value*
Surgical Treatment	1. Lumpectomy or partial mastectomy	15556	1131 (7.3%)	742 (7.1%)	389 (7.6%)	**0.014**
	2. Total mastectomy		2524 (16.2%)	1756 (16.8%)	768 (15.0%)	
	3. No surgery		11901 (76.5%)	7950 (76.1%)	3951 (77.3%)	
Hormone Treatment	1. Yes	15556	11546 (74.2%)	8078 (77.3%)	3468 (67.9%)	**<.001**
	2. No		4010 (25.8%)	2370 (22.7%)	1640 (32.1%)	
Chemotherapy	1. Yes	15556	8880 (57.1%)	5778 (55.3%)	3102 (60.7%)	**<.001**
	2. No		6676 (42.9%)	4670 (44.7%)	2006 (39.3%)	
Comorbidity Score	0	15556	12337 (79.3%)	8320 (79.6%)	4017 (78.6%)	0.164
	1		2241 (14.4%)	1484 (14.2%)	757 (14.8%)	
	2		653 (4.2%)	442 (4.2%)	211 (4.1%)	
	>=3		325 (2.1%)	202 (1.9%)	123 (2.4%)	
Year of Diagnosis	2010	15556	1225 (7.9%)	855 (8.2%)	370 (7.2%)	**<.001**
	2011		1540 (9.9%)	1082 (10.4%)	458 (9.0%)	
	2012		1637 (10.5%)	1187 (11.4%)	450 (8.8%)	
	2013		1783 (11.5%)	1223 (11.7%)	560 (11.0%)	
	2014		2039 (13.1%)	1364 (13.1%)	675 (13.2%)	
	2015		2147 (13.8%)	1442 (13.8%)	705 (13.8%)	
	2016		2488 (16.0%)	1623 (15.5%)	865 (16.9%)	
	2017		2697 (17.3%)	1672 (16.0%)	1025 (20.1%)	

*p-value was calculated using Chi-Square tests.

Supplementary Figure 1A. Survival Curve by HER2 Expression Level(IHC0, IHC1+, IHC2+) and Hormonal Receptor Status

HR+ Breast Cancer (HER2 IHC2/ISH Neg vs IHC 1+ vs IHC 0)

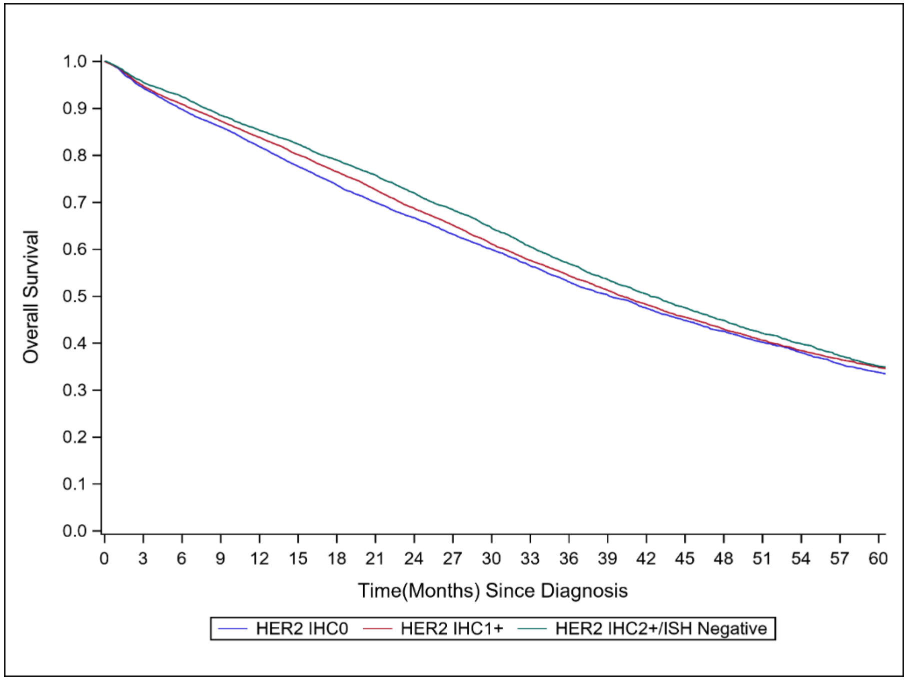

HR- Breast Cancer (HER2 IHC2/ISH Neg vs IHC 1+ vs IHC 0)

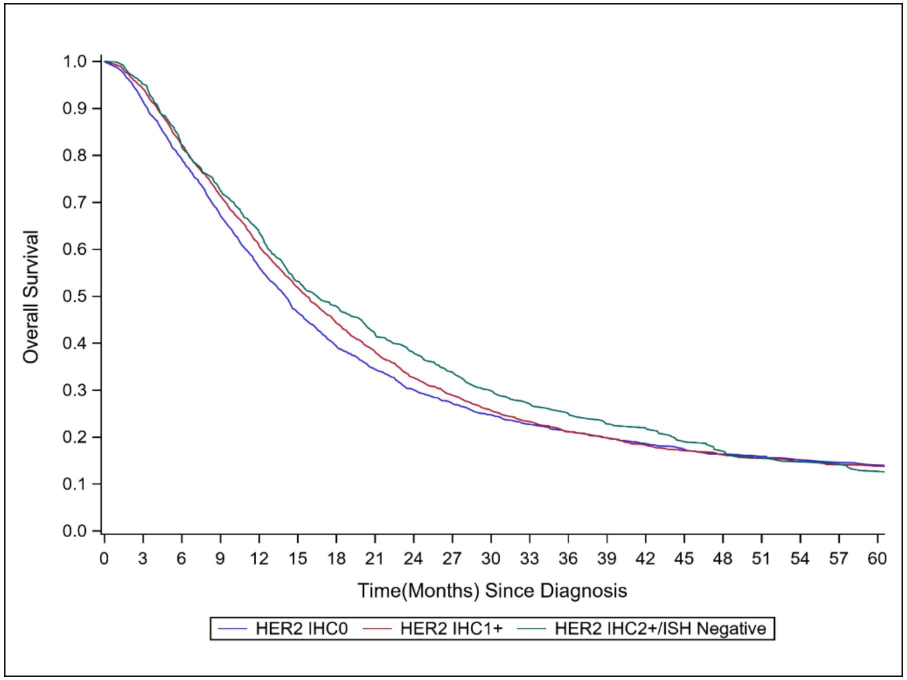

Supplementary Figure 1B. Survival Curve by HER2 Expression Level(IHC0, IHC1+, IHC2+) and Patients' Race

All Breast Cancer (White) (HER2 IHC2/ISH Neg vs IHC 1+ vs IHC 0)

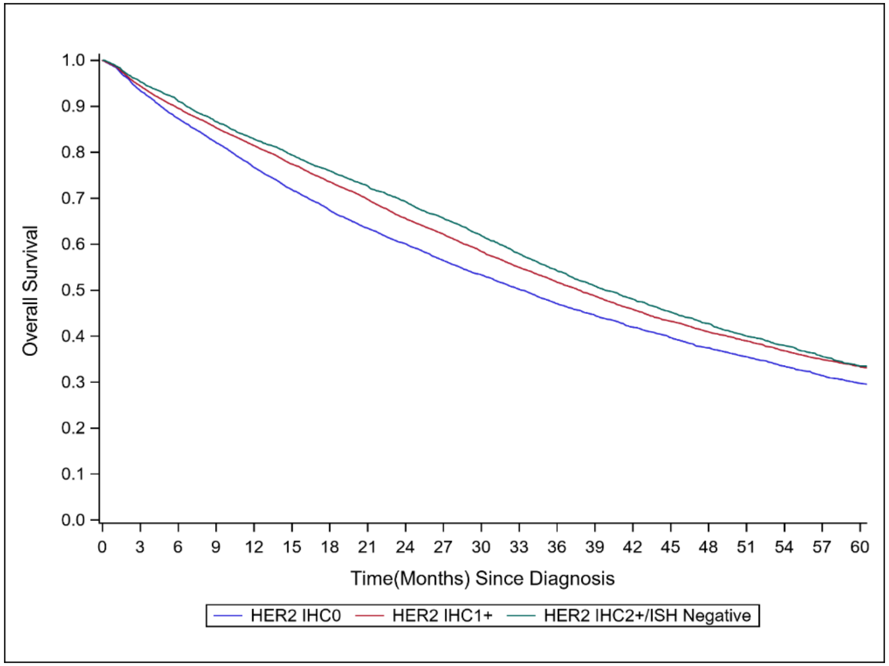

All Breast Cancer (Non-White) (HER2 IHC2/ISH Neg vs IHC 1+ vs IHC 0)

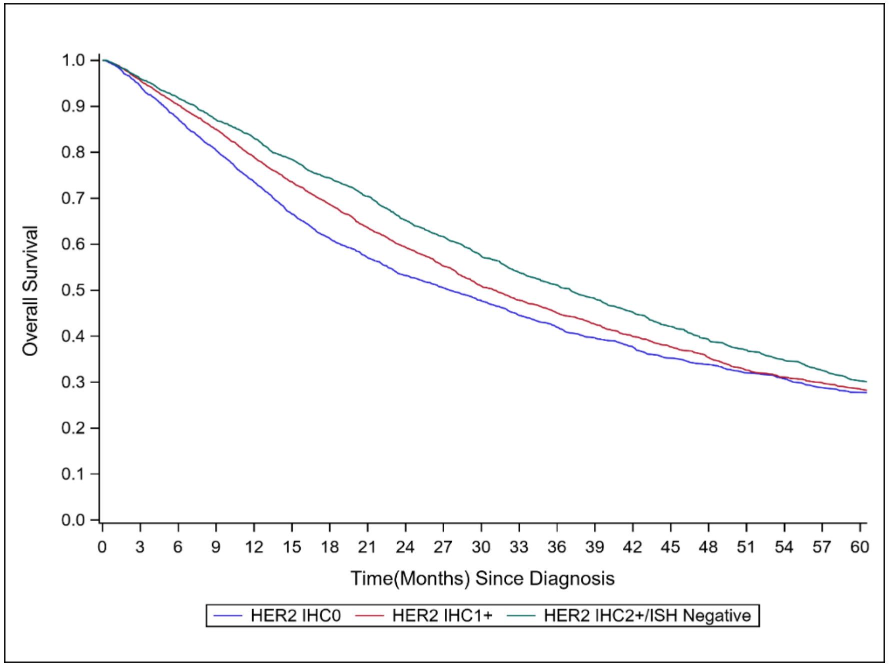

Supplementary Figure 1C. Survival Curve by HER2 Expression Level(IHC0, IHC1+, IHC2+) and Type of Treatment

All Breast Cancer, Receipt of Chemotherapy
(HER2 IHC2/ISH Neg vs IHC 1+ vs IHC 0)

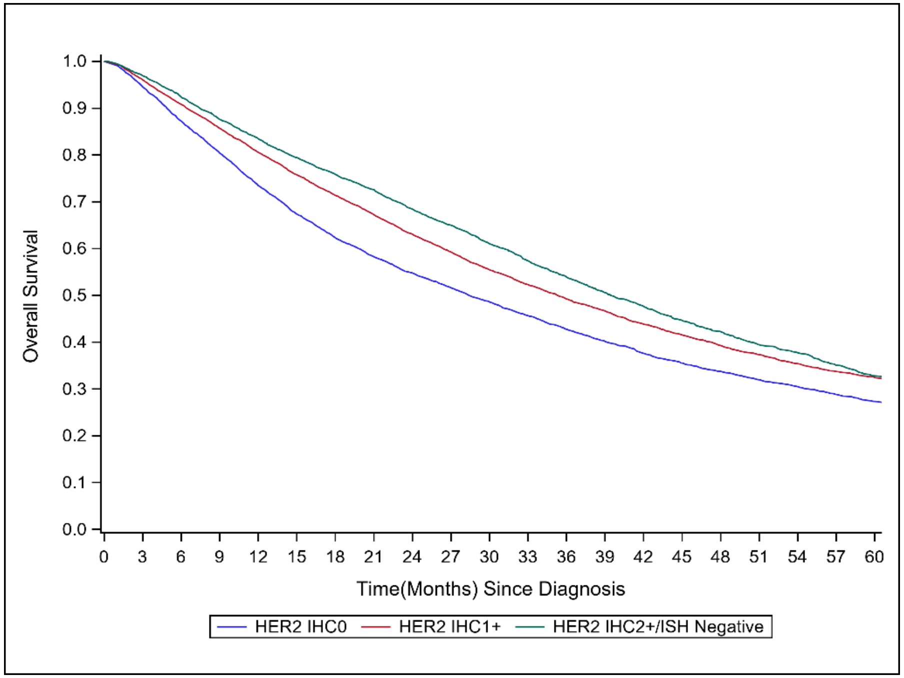

All Breast Cancer, Receipt of Hormonal Therapy Only
(HER2 IHC2/ISH Neg vs IHC 1+ vs IHC 0)

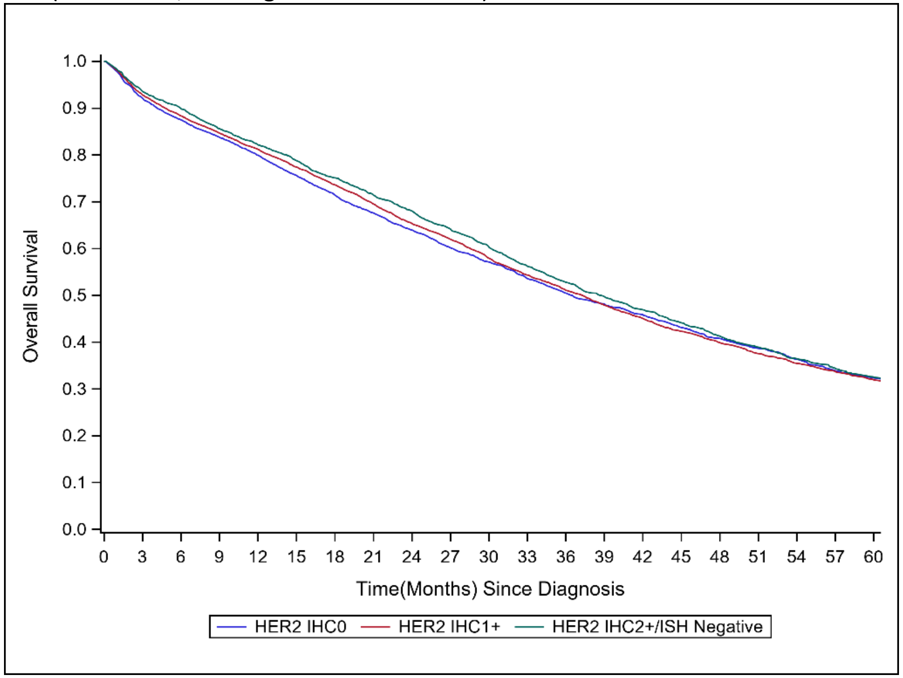

Supplementary Figure 2A - Adjusted Hazard Ratio (HER2-low vs. HER2-zero breast cancer) from Multivariable Cox regression analysis for Overall Survival
(Sensitivity analysis in patients whose initial diagnosis and first course treatment given at the same reporting facility.).

Subgroup	HER2 Low Events/N	(%)	HER2 Zero Events/N	(%)	HR (95% CI)	P Value*
**Overall**	6653/10448	(63.7)	3328/5108	(65.2)	0.93 (0.89, 0.97)	
**Age**						0.09
<55y	1658/2772	(59.8)	913/1493	(61.2)	1.00 (0.92, 1.09)	
≥55y	4995/7676	(65.1)	2415/3615	(66.8)	0.91 (0.87, 0.96)	
**Race**						0.21
White	4821/7628	(63.2)	2364/3639	(65.0)	0.92 (0.87, 0.96)	
Non-White	1832/2820	(65.0)	964/1469	(65.6)	0.97 (0.89, 1.05)	
**Tumor Grade**						0.20
Well/Moderated Differentiated	3018/5136	(58.8)	1176/2087	(56.3)	0.96 (0.90, 1.03)	
Poorly Differentiated	3635/5312	(68.4)	2152/3021	(71.2)	0.91 (0.86, 0.96)	
**Histology**						0.03
Ductal	4371/6966	(62.7)	2099/3207	(65.5)	0.89 (0.85, 0.94)	
Non-Ductal/Unknown	2282/3482	(65.5)	1229/1901	(64.7)	1.00 (0.93, 1.07)	
**Visceral Metastases at Diagnosis**						0.51
Yes	3172/4501	(70.5)	1670/2308	(72.4)	0.92 (0.87, 0.98)	
No	3481/5947	(58.5)	1658/2800	(59.2)	0.94 (0.88, 1.00)	
**Systemic Therapy**						0.03
Any Chemotherapy	3483/5778	(60.3)	2007/3102	(64.7)	0.89 (0.84, 0.94)	
Only Hormonal Therapy	3170/4670	(67.9)	1321/2006	(65.9)	0.98 (0.92, 1.04)	

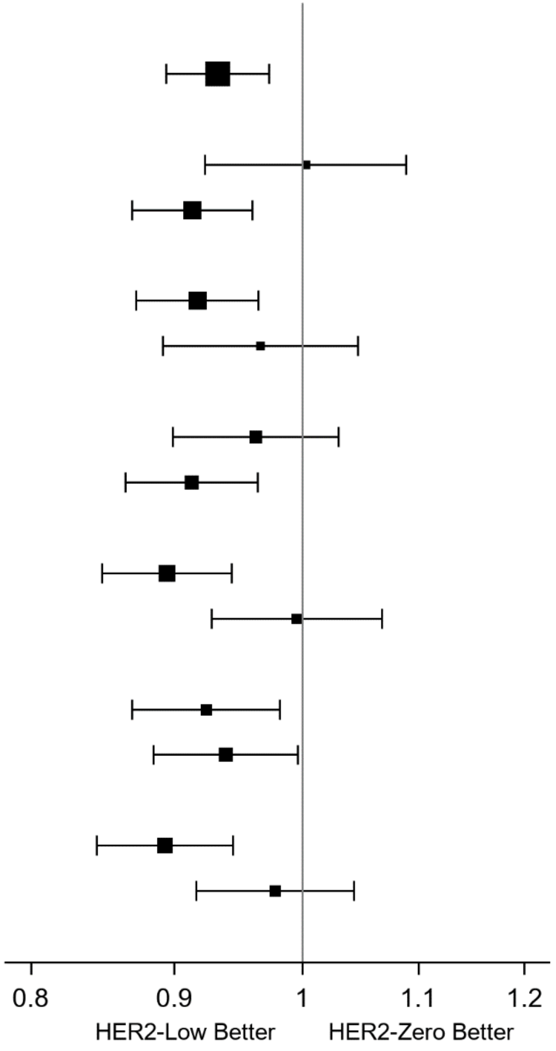

* P-Value is the statistical test of interaction between HER2 expression level and each subgroup.

Supplementary Figure 2B - Adjusted Hazard Ratio (HER2-low vs. HER2-zero breast cancer) in HR+ Breast Cancer from Multivariable Cox regression analysis for Overall Survival
(Sensitivity analysis in patients whose initial diagnosis and first course treatment given at the same reporting facility.).

Subgroup	HER2 Low Events/N	(%)	HER2 Zero Events/N	(%)	HR (95% CI)	P Value*
**Overall**	5655/9191	(61.5)	2397/3970	(60.4)	0.95 (0.91, 1.00)	
**Age**						0.31
<55y	1301/2316	(56.2)	579/1067	(54.3)	1.01 (0.91, 1.11)	
≥55y	4354/6875	(63.3)	1818/2903	(62.6)	0.94 (0.89, 0.99)	
**Race**						0.43
White	4207/6856	(61.4)	1788/2949	(60.6)	0.94 (0.89, 0.99)	
Non-White	1448/2335	(62.0)	609/1021	(59.6)	0.98 (0.89, 1.08)	
**Tumor Grade**						0.17
Well/Moderated Differentiated	2846/4911	(58.0)	1051/1934	(54.3)	0.99 (0.92, 1.07)	
Poorly Differentiated	2809/4280	(65.6)	1346/2036	(66.1)	0.92 (0.86, 0.98)	
**Histology**						0.01
Ductal	3639/6032	(60.3)	1428/2365	(60.4)	0.90 (0.85, 0.96)	
Non-Ductal/Unknown	2016/3159	(63.8)	969/1605	(60.4)	1.03 (0.95, 1.11)	
**Visceral Metastases at Diagnosis**						0.83
Yes	2594/3802	(68.2)	1076/1611	(66.8)	0.96 (0.89, 1.03)	
No	3061/5389	(56.8)	1321/2359	(56.0)	0.95 (0.89, 1.01)	
**Systemic Therapy**						0.14
Any Chemotherapy	2508/4548	(55.1)	1094/1985	(55.1)	0.91 (0.85, 0.98)	
Only Hormonal Therapy	3147/4643	(67.8)	1303/1985	(65.6)	0.98 (0.92, 1.05)	

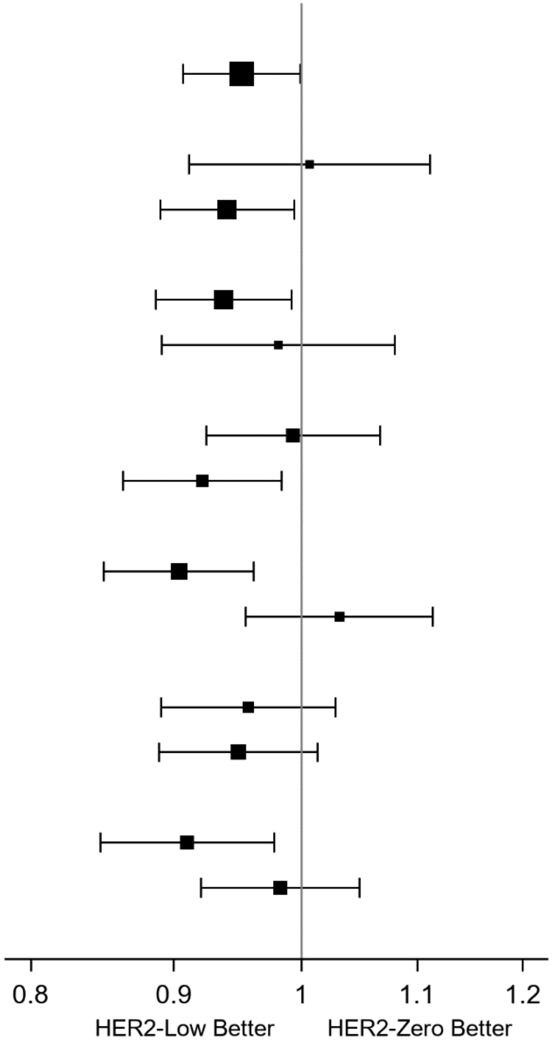

* P-Value is the statistical test of interaction between HER2 expression level and each subgroup.

Supplementary Figure 2C - Adjusted Hazard Ratio (HER2-low vs. HER2-zero breast cancer) in HR- Breast Cancer from Multivariable Cox regression analysis for Overall Survival
(Sensitivity analysis in patients whose initial diagnosis and first course treatment given at the same reporting facility.).

Subgroup	HER2 Low Events/N (%)		HER2 Zero Events/N (%)		HR (95% CI)	P Value*
**Overall**	998/1257	(79.4)	931/1138	(81.8)	0.86 (0.79, 0.94)	
**Age**						0.05
<55y	357/456	(78.3)	334/426	(78.4)	0.98 (0.84, 1.14)	
≥55y	641/801	(80.0)	597/712	(83.8)	0.81 (0.73, 0.91)	
**Race**						0.13
White	614/772	(79.5)	576/690	(83.5)	0.81 (0.72, 0.91)	
Non-White	384/485	(79.2)	355/448	(79.2)	0.94 (0.81, 1.09)	
**Tumor Grade**						0.28
Well/Moderated Differentiated	172/225	(76.4)	125/153	(81.7)	0.75 (0.59, 0.97)	
Poorly Differentiated	826/1032	(80.0)	806/985	(81.8)	0.89 (0.80, 0.98)	
**Histology**						0.67
Ductal	732/934	(78.4)	671/842	(79.7)	0.86 (0.77, 0.96)	
Non-Ductal/Unknown	266/323	(82.4)	260/296	(87.8)	0.82 (0.68, 0.98)	
**Visceral Metastases at Diagnosis**						0.52
Yes	578/699	(82.7)	594/697	(85.2)	0.84 (0.75, 0.95)	
No	420/558	(75.3)	337/441	(76.4)	0.90 (0.77, 1.04)	

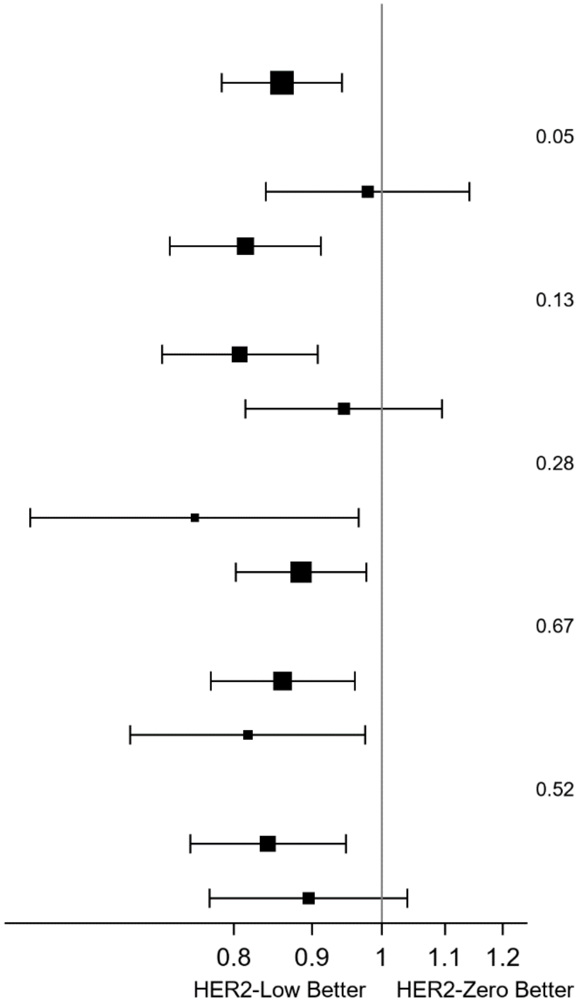

* P-Value is the statistical test of interaction between HER2 expression level and each subgroup.
