# Supplementary material for: Clinical outcomes of de novo metastatic HER2-low breast cancer: a National Cancer Database Analysis
Source: NPJ Breast Cancer. 2022 Dec 30;8:135. doi: 10.1038/s41523-022-00498-8 (PMC9803673; doi:10.1038/s41523-022-00498-8)
Supplement: Supplementary file 2 — Supplementary Figures [file 41523_2022_498_MOESM2_ESM.docx]

**Supplementary Figure1 . Survival Curve by Level of HER2 Expression Level(IHC0, IHC1+, IHC2+) and Socio-clinical Factors**

A. By Hormonal Receptor Status: Left: HR+ Breast Cancer (HER2 Low vs HER2 Zero); Right: HR- Breast Cancer (HER2 Low vs HER2 Zero)

B. By Patients’ Race: Left: HR+ Breast Cancer (White); Right: HR- Breast Cancer (Non-white)

C. By Type of Treatment : Left: All Breast Cancer, Receipt of Chemotherapy; Right: All Breast Cancer, Receipt of Hormonal Therapy Only

Log-rank test was used to compare the survival across HER2 expression.

**Supplementary Figure2- Adjusted Hazard Ratio (HER2-low vs. HER2-zero breast cancer) from Multivariable Cox regression analysis for Overall Survival (Sensitivity analysis in patients whose initial diagnosis and first course treatment given at the same reporting facility. ).**

A: All breast cancer; B: HR+ Breast Cancer; C: HR- Breast Cancer

p-value is calculated for interaction between HER2 expression level and each subgroup, based on using multivariable cox regression. Error bars showed the confidence interval of hazard ratio of OS between HER2-low vs HER2-zero breast cancer.

The cox regression models were adjusted for age, race/ethnicity, household income, comorbidities, location, tumor grade, histology, hormonal receptor status (if applicable), presence of visceral metastasis, the type of cancer center (where women received care), year of diagnosis, and treatment type.
